# Hsa_Circ_0001947/MiR-661/DOK7 Axis Restrains Non-Small Cell Lung Cancer Development

**DOI:** 10.4014/jmb.2106.06031

**Published:** 2021-09-11

**Authors:** Yuyan Bao, Yanjie Yu, Bing Hong, Zhenjian Lin, Guoli Qi, Jie Zhou, Kaiping Liu, Xiaomin Zhang

**Affiliations:** 1 Pharmaceutical Preparation Section, Sanmen People's Hospital, 15 Taihe Road, Hairun Street, Sanmen County, Zhejiang Province, 317100, P.R.China; 2 Sanmen Market Supervisory Authority, 5 Qiushui Avenue, Haiyou Town, Sanmen County, Zhejiang Province, 317100, P.R.China

**Keywords:** Non-small cell lung cancer, hsa_circ_0001947, miR-661, downstream of tyrosine kinase 7

## Abstract

Hsa_circ_0001947 is associated with multiple cancers, but its function in non-small cell lung cancer (NSCLC) is ambiguous and needs further research. The targeting relationship among circ_0001947, miR-661, and downstream of tyrosine kinase 7 (DOK7) was predicted by database and further verified by dual-luciferase reporter assay, while their expressions in cancer tissues and cells were detected by quantitative real-time polymerase chain reaction (qRT-PCR). After transfection, cell biological behaviors and expressions of miRNAs, miR-661 and DOK7 were determined by cell function experiments and qRT-PCR, respectively. Circ_0001947 was low-expressed in NSCLC tissues and cells. Circ_0001947 knockdown intensified cell viability and proliferation, induced cell cycle arrest at S phase, suppressed apoptosis and evidently enhanced miR-510, miR-587, miR-661 and miR-942 levels, while circ_0001947 overexpression did the opposite. MiR-661 was a target gene of circ_0001947 that participated in the regulation of circ_0001947 on cell biological behaviors. Furthermore, DOK7, the target gene of miR-661, partly participated in the regulation of miR-661 on cell viability. Hsa_circ_0001947 acts as a sponge of miR-661 to repress NSCLC development by elevating the expression of DOK7.

## Introduction

Lung cancer is a prevalent malignancy in the world. According to statistics, there were approximately 2.1 million new cases and 1.8 million deaths worldwide in 2018, ranking first among all cancer types [1]. Non-small cell lung cancer (NSCLC) is the most common histopathological subtype of lung cancer, accounting for 80-90% of lung cancer [2]. NSCLC is generally diagnosed at an advanced stage, and 90% of patients die from distant metastasis [3]. The five-year survival rate of patients with NSCLC could be improved to 50%-70% with early diagnosis and treatment prior to metastasis of the disease, otherwise the rate may be less than 5% [4]. In addition, the lack of relatively specific tumor markers poses challenges to the diagnosis, treatment, and prognosis of NSCLC. Therefore, it is extremely important and necessary to find avenues that are conducive to early diagnosis and treatment of NSCLC.

CircRNA is a special endogenous non-coding RNA that has long been found in RNA viruses [5]. However, due to technical limitations at that time, circRNA was considered a by-product of the splicing process and thus received little interest [6]. In recent years, with the development of sequencing technology, circRNA has been found to be widely presented and stably expressed in eukaryotic cells, playing a pivotal role in the regulation of gene expression in human cells [7]. Current researches pointed out that circRNA may serve as a molecular marker for prognostic judgment of different tumors including NSCLC [8, 9]. Studies have manifested that some circRNAs can participate in the pathogenesis of NSCLC as oncogenes or tumor suppressor genes, such as circ_BANP, circ_100395, and circ_0001947 [10-12]. Among them, only one research proposed that circ_0001947 may be closely associated with the presence and progression of NSCLC [13]. However, the exact role of circ_0001947 in NSCLC has not been reported yet.

Moreover, it has been put forward that multiple circRNAs themselves contain at least one miRNA binding site, which can act as the “sponge” of RNA to adsorb miRNA, thus regulating miRNA-induced inhibition of downstream target genes expressions through the mechanism of competitive endogenous RNAs (ceRNAs) [14]. MiRNA controls gene expression by binding to the 3’-UTR of target gene mRNA, resulting in target gene translation inhibition or degradation [15]. More and more evidence exhibited that a series of miRNAs (miR-96-5p, miR-133a-5p, miR-182-5p, and miR-661) are closely related to the progression of NSCLC [16-19]. Gu *et al*. confirmed that GAPLINC accelerates the progression of NSCLC via modulating the miR-661/eEF2K axis [19]. Nevertheless, the participation of circ_0001947 in NSCLC progression via modulating miR-661 remains to be clarified.

Based on the above evidence, we aimed to figure out the influence of the circ_0001947/miR-661 axis on the biological behaviors of NSCLC cells and to explore whether circ_0001947 and miR-661 have complementary binding sites to determine potential therapeutic targets for NSCLC.

## Materials and Methods

### Ethics Statement

We obtained NSCLC tissues and adjacent non-tumor tissues from 40 patients who had been diagnosed and operated in the Sanmen Peoplés Hospital between March 2018 and July 2019. All participants signed written informed consent. The present research was ratified by the Ethics Committee of the Sanmen Peoplés Hospital (2018020039).

In this research, tissue specimens were acquired via surgical resection and snap-frozen in a -80°C refrigerator (TSE600V, Thermo Scientific, USA). We used the quantitative real-time polymerase chain reaction (qRT-PCR) to examine the circ_0001947, miR-661 and DOK7 expressions.

### Cell Culture

Four human NSCLC cell lines H1299 (CRL-5803), A549 (CCL-185), H358 (CRL-5807), and H1395 (CRL-5868) were obtained from American Type Culture Collection (ATCC, USA). A human bronchial epithelial cell line (16HBE, MXC003) was procured from MEIXUAN (China). The above cells were grown in RPMI 1640 medium (30-2001, ATCC) supplemented with 10% fetal bovine serum (FBS, 30-2020, ATCC) in a Nunc Cell Factory Incubator (120300, Thermo Scientific) at 37°C with 5% CO_2_.

### Cell Transfection

Small interfering RNAs targeting circ_0001947 (si-circ_0001947#1, sequence: 5’-GATGGACATGTAAACAAATTT-3’), si-circ_0001947#2 (sequence: 5’- TGGAACAGAGTCTACAAATAA-3’) and downstream of tyrosine kinase 7 (siDOK7, sequence: 5’-GACTGGAAATAAAATGTTCTTTC-3’), and non-targeting negative control (si-NC, sequence: 5’-ACCCGTTTCCGATTCTTTTAGTT-3’) were constructed by YouBio and inserted into the pSilencer 4.1-CMV neo vector (VT1395, YouBio, China). The full-length sequences of circ_0001947 and DOK7 were constructed from Invitrogen and inserted into the pcDNA3.1 vector (V87020, Invitrogen, China). Empty vector was used as the NC. MiR-661 mimic (M, miR10003324-1-5), inhibitor (I, miR20003324-1-5), mimic control (MC, miR1N0000001-1-5), and inhibitor control (IC, miR2N0000001-1-5) were obtained from RiboBio (China). We used the lipofectamine 2000 (12566014, Invitrogen, USA) to transfect the above plasmids into A549 and H1299 cells. After 48 h (h) of transfection, qRT-PCR was used to determine the transfection rate.

### Cell Processing

First, to fathom out the role of circ_0001947 on cells, the cells were assigned into the control group, NC group, circ_0001947 group, si-NC group, and si-circ_0001947#2 group. In the last four groups, cells were transfected with NC, overexpressed circ_0001947, si-NC and si-circ_0001947#2, respectively. Second, to ascertain the functions of si-circ_0001947#2 and miR-661 I on cells, the cells were separated into the control group, si-NC + IC group (co-transfection with si-NC and miR-661 IC), si-circ_0001947#2 + IC group (co-transfection with si-circ_0001947#2 and miR-661 IC), si-circ_0001947#2 + I group (co-transfection with si-circ_0001947#2 and miR-661 I), and si-NC + I group (co-transfection with si-NC and miR-661 I). Third, to investigate the functions of overexpressed circ_0001947 and miR-661 M on cells, cells were divided into the control group, NC + MC group (co-transfection with NC and miR-661 MC), circ_0001947 + MC group (co-transfection with overexpressed circ_0001947 and miR-661 MC), circ_0001947 + M group (co-transfection with overexpressed circ_0001947 and miR-661 M), and NC + M group (co-transfection with NC and miR-661 M). Fourth, to comprehend the roles of miR-661 I and siDOK7 on cells, cells were classified into the IC + siNC group (co-transfection with miR-661 IC and siNC), I + siNC group (co-transfection with miR-661 I and siNC), I + siDOK7 group (co-transfection with miR-661 I and siDOK7), and IC + siDOK7 group (co-transfection with miR-661 IC and siDOK7). Fifth, to investigate the functions of miR-661 M and overexpressed DOK7 on cells, cells were divided into the MC + NC group (co-transfection with miR-661 MC and NC), M + NC group (co-transfection with miR-661 M and NC), M+ DOK7 group (co-transfection with miR-661 M and DOK7), and MC + DOK7 group (co-transfection with miR-661 MC and overexpressed DOK7).

### qRT-PCR

RNA from lung tissues and cells was acquired using the RNA Extraction Kit (N065, China). Next, to quantify the mRNA expression, cDNA synthesis was conducted in line with instructions of the cDNA first chain synthesis kit (N118, China) and miRNA cDNA first-strand synthesis kit (KR211, China), respectively. Then, cDNA was amplified and quantitatively analyzed by the FastFire qPCR PreMix (FP207-02, Tiangen) in a C1000 Touch PCR system (E1138, Bio-Rad, USA). GAPDH and U6 were employed for normalization controls. The results were normalized by the 2^-ΔΔCt^ method [20]. The primers were listed in Table 1.

### Cell Counting Kit-8 (CCK-8)

We used the CCK-8 kit (abs50003, absin, China) to assess cell viability. Based on the above groups, cells (1 × 10^4^ per well) were transfected and then maintained in an incubator for a specified time (24 or 48 h). Next, 10 μl CCK-8 solution was adopted to treat cells for 4 h. Then, the optical density at a wavelength of 450 nm was determined under a microplate reader (Z742711, Sigma, USA).

### Apoptosis Analysis

The measurement of cell apoptosis was performed using the Annexin V-FITC/propidium iodide (PI) kit (C1062S, Beyotime, China). Cells (5 × 10^4^ per well) were treated according to the above grouping conditions. After treatment, 195 μl binding buffer was added to the cell suspensions. Thereafter, 5 μl Annexin V-FITC and 10 μl PI were added to the cell suspensions and mixed well. The mixture was placed in an incubator at 37°C for 15 min (min). Finally, we used a flow cytometer (DxFLEX, Beckman, USA) to examine the FITC and PI signals.

### Colony Formation Assay

In this research, the cells in good growth condition were made into a single-cell suspension. Cell suspension (1×10^4^ cells/ml) was seeded into a 6-well plate and then transfected based on the above groups. After incubation for 14 days, we used the 4% paraformaldehyde (P0099, Beyotime) and Giemsa dye solution (G8220, Solarbio, China) to fix and stain cells, respectively. Finally, we used a microscope (Z723975-1EA, Sigma) to count the clone numbers of the cells.

### Cell Cycle Assay

Cell Cycle and Apoptosis Analysis Kit (C1052, Beyotime) was used in this research. After treatment, cells (1×10^5^ cells/ml) were collected and centrifuged. After being washed, the cells were immobilized by 70% cold ethanol solution (E111991, Aladdin, China) at 4°C overnight. Then, the cells were washed and reacted with prepared 0.5 ml PI stain solution at 37°C in the dark for 30 min. Finally, the percentage of NSCLC cells at different stages of cell cycle was examined using flow cytometer.

### Target Gene Prediction and Dual-Luciferase Reporter Assay

Binding sites between circ_0001947 and miR-661 as well as between miR-661 and DOK7 were determined by CircInteractome (https://circinteractome.irp.nia.nih.gov/) or TargetScan v7.2 (https://www.targetscan.org/). The wild-type (WT) or mutated (MUT) circ_0001947 and DOK7 sequences were amplified by PCR and inserted into the pmirGLO vector (LM1439, LMAI Bio, China) to construct circ_0001947-WT, circ_0001947-MUT, DOK7-WT, and DOK7-MUT report plasmids. Then, all report plasmids were co-transfected with miR-661 M or MC into cells by lipofectamine 2000. The role of miR-661 M on luciferase activity was assessed after 48 h of transfection using the luciferase assay kit (16181, Thermo Scientific) under the chemiluminescence instrument (GloMax 2020, Promega, USA).

### Statistical Analysis

Data were gathered and analyzed by GraphPad Prism 8.0 (GraphPad Software Inc., USA). The measurement data were expressed as mean ± standard deviation (SD). The levels of circ_0001947, miR-661, and DOK7 in 40 NSCLC tissues and adjacent non-tumor tissues were analyzed by paired-samples *t* test. An independent sample t test was employed to compare the difference of two groups. The multiple group comparisons were completed through a one-way analysis of variance (ANOVA) followed by Tukey’s post hoc test. *p* < 0.05 was considered as statistically significant.

## Results

### Circ_0001947 Was Low-Expressed in NSCLC Tissues and Cells and Si-Circ_0001947#2 Intensified the NSCLC Cell Viability

**Fig. 1:**
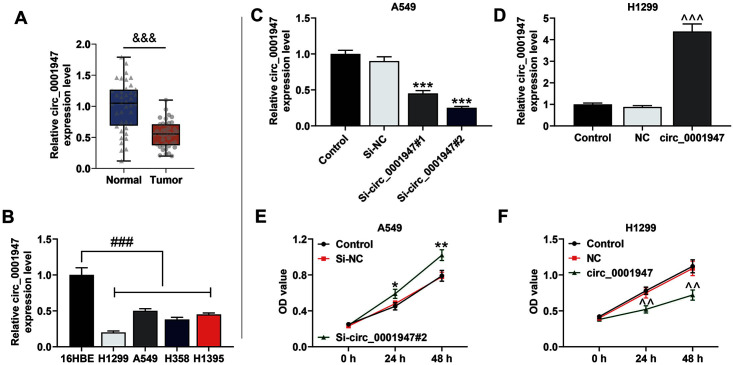
Circ_0001947 was low-expressed in NSCLC tissues and cells and si-circ_0001947#2 intensified the NSCLC cell viability. **A**. The level of circ_0001947 in 40 non-small cell lung cancer (NSCLC) tissues and adjacent nontumor tissues was analyzed by quantitative real-time polymerase chain reaction (qRT-PCR). Glyceraldehyde-3-phosphate dehydrogenase (GAPDH) was used as the internal control. (^&&&^*p* < 0.001 vs. normal) **B**. The level of circ_0001947 in NSCLC cells and normal cells was analyzed by qRT-PCR. GAPDH was used as the internal control. (^###^*p* < 0.001 vs.16HBE) **C-D**. The level of circ_0001947 in NSCLC cells transfected with small interfering RNA targeting circ_0001947 (si-circ_0001947#1), sicirc_ 0001947#2, or overexpressed circ_0001947 was analyzed by qRT-PCR. GAPDH was used as the internal control. (***p < 0.001 vs. small interfering RNA targeting negative control (si-NC) group; ^^^*p* < 0.001 vs. negative control (NC) group) **E-F**. The viability of NSCLC cells transfected with si-circ_0001947#2 or overexpressed circ_0001947 was analyzed by Cell Counting Kit- 8 (CCK-8). (*p < 0.05, **p < 0.01 vs. si-NC group; ^^*p* < 0.01 vs. NC group) All experiments have been performed in triplicate and data were expressed as mean ± standard deviation (SD).

To explore the level of circ_0001947 in NSCLC tissues or cell lines, we checked the data from qRT-PCR and clarified that circ_0001947 expression was lower in NSCLC tissues than that in adjacent non-tumor tissues (Fig. 1A), while the same trend was discovered in NSCLC cells when compared with that in 16HBE cells (Fig. 1B). Since circ_0001947 expression was notably down-regulated in H1299 cells and slightly restrained in A549 cells relative to other NSCLC cells, H1299 and A549 cells were singled out for subsequent experiments. Next, we transfected si-circ_0001947#1, si-circ_0001947#2, circ_0001947 overexpression plasmids, or their NCs into H1299 and A549 cells, confirming that circ_0001947 overexpression plasmids largely enhanced the circ_0001947 level, while si-circ_0001947#1 and si-circ_0001947#2 showed opposite results (Figs. 1C-1D). Since si-circ_0001947#2 generated a stronger inhibitory effect on the circ_0001947 level relative to the si-circ_0001947#1, we chose si-circ_0001947#2 for the following experiments. Then, the CCK-8 assay manifested that the cell viability was mightily strengthened by si-circ_0001947#2 but notably repressed by overexpressed circ_0001947 (Figs. 1E and 1F).

### Si-circ_0001947#2 Intensified Cell Proliferation, Induced Cell Cycle Arrest at S Phase and Restrained Apoptosis, while Overexpressed circ_0001947 did the Opposite

**Fig. 2:**
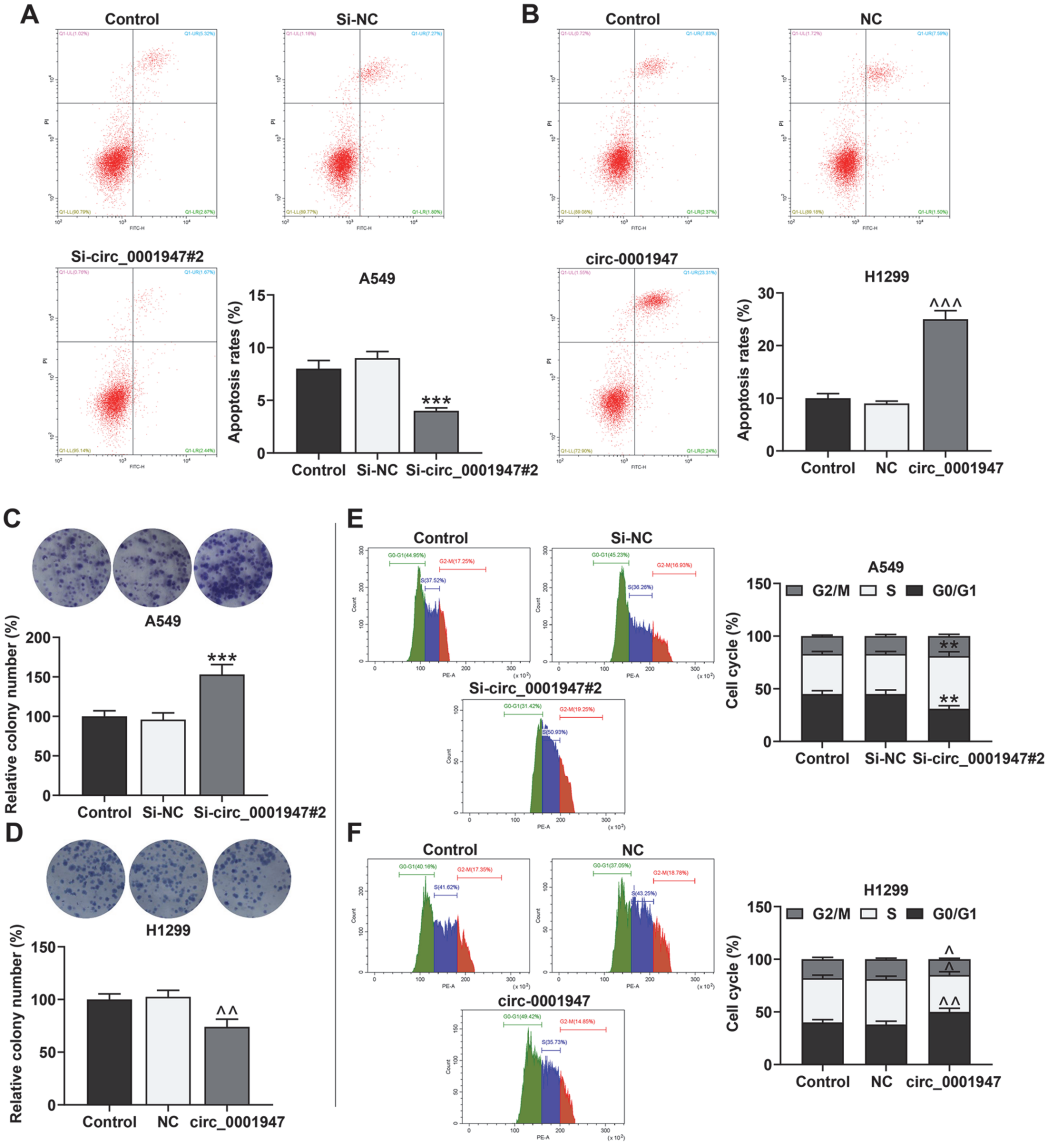
Si-circ_0001947#2 intensified cell proliferation, induced cell cycle arrest at S phase and restrained apoptosis, while overexpressed circ_0001947 did the opposite. **A-B**. Apoptosis of NSCLC cells transfected with sicirc_ 0001947#2 or overexpressed circ_0001947 was analyzed by flow cytometry. (***p < 0.001 vs. si-NC group; ^^^*p* < 0.001 vs. NC group) **C-D**. Proliferation of NSCLC cells transfected with si-circ_0001947#2 or overexpressed circ_0001947 was analyzed by colony formation assay. (***p < 0.001 vs. si-NC group; ^^*p* < 0.01 vs. NC group) **E-F**. Cell cycle of NSCLC cells transfected with si-circ_0001947#2 or overexpressed circ_0001947 was analyzed by flow cytometry. (**p < 0.01 vs. si-NC group; ^*p* < 0.05, ^^*p* < 0.01 vs. NC group)

The flow cytometer and colony formation assay exhibited that si-circ_0001947#2 markedly restrained apoptosis and enhanced cell proliferation, while overexpressed circ_0001947 generated the inverse effects (Figs. 2A-2D). Moreover, si-circ_0001947#2 induced cell cycle arrest at S phase whereas circ_0001947 overexpression facilitated the entry of G0/G1 phase but restrained the entry of G2/M and S phases (Figs. 2E and 2F).

### Si-circ_0001947#2 Enhanced the miR-510, miR-587, miR-661 and miR-942 Levels and circ_0001947 Targeted miR-661

**Fig. 3:**
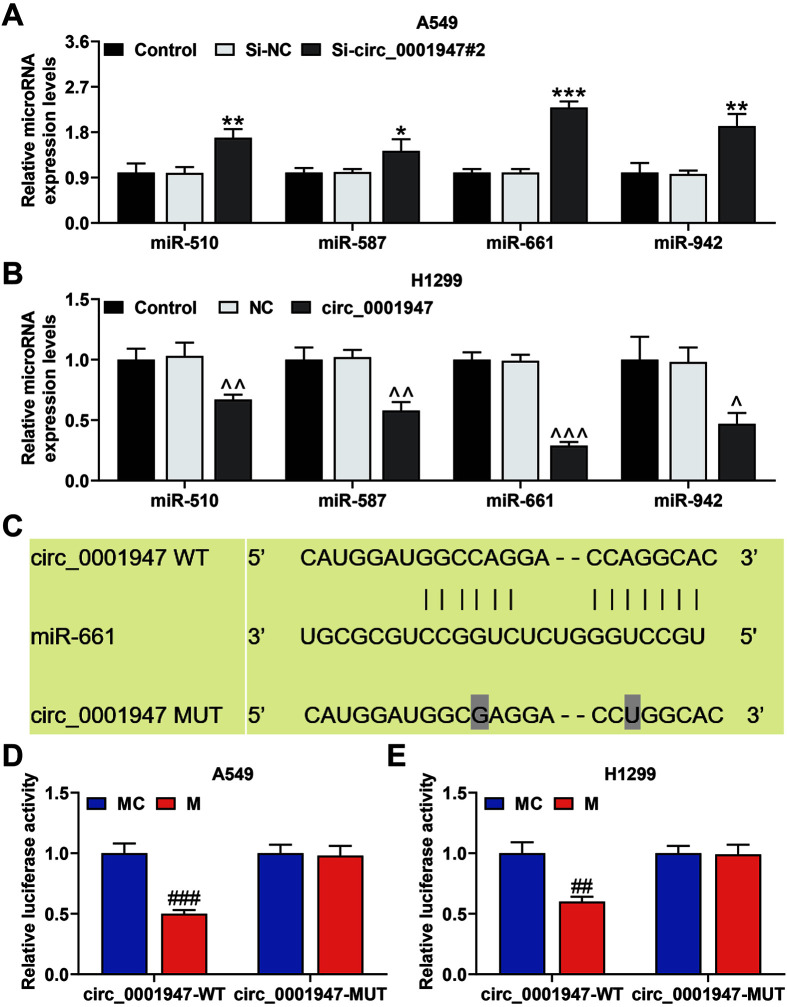
Si-circ_0001947#2 enhanced miR-510, miR-587, miR-661, and miR-942 levels and circ_0001947 targeted miR-661. **A-B**. The levels of miR-510, miR-587, miR-661, and miR-942 in NSCLC cells transfected with sicirc_ 0001947#2 or overexpressed circ_0001947 were analyzed by qRT-PCR. U6 was used as the internal control. (*p < 0.05, **p < 0.01, ***p < 0.001 vs. si-NC group; ^*p* < 0.05, ^^*p* < 0.01, ^^^*p* < 0.001 vs. NC group) **C**. The binding sequences of circ_0001947 containing the miR-661 binding sites were determined by CircInteractome (https://circinteractome.irp.nia.nih.gov/). **D-E**. Direct interaction between circ_0001947 and miR-661 was detected by dual-luciferase reporter assay. (^##^*p* < 0.01, ^###^*p* < 0.001 vs. mimic control (MC) group)

The online database CircInteractome helped us acquire 47 miRNAs interacting with circ_0001947. We finally detected the levels of these miRNAs related to NSCLC and speculated to bind to circ_0001947, including miR-510, miR-587, miR-661 and miR-942. The qRT-PCR assay corroborated that si-circ_0001947#2 evidently enhanced the miR-510, miR-587, miR-661, and miR-942 levels, while circ_0001947 overexpression did the opposite (Figs. 3A and 3B). Since si-circ_0001947#2 and circ_0001947 overexpression exerted the most significant effects on the level of miR-661, miR-661 was screened out for subsequent experiments. CircInteractome analysis was used to certify that circ_0001947 targeted miR-661 (Fig. 3C). Since then, we further confirmed that miR-661 M greatly reduced the luciferase activity of circ_0001947-WT rather than that of circ_0001947-MUT in H1299 and A549 cells (Figs. 3D and 3E).

### MiR-661 Was Overexpressed in NSCLC Tissues and Cells, and Participated in the Regulation of Circ_0001947 on Cell Viability

**Fig. 4:**
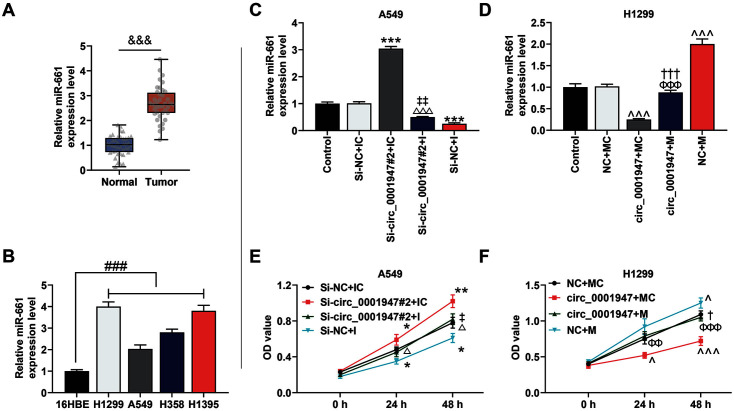
MiR-661 was overexpressed in NSCLC tissues and cells and participated in the regulation of circ_0001947 on cell viability. **A**. The level of miR-661 in 40 NSCLC tissues and adjacent non-tumor tissues was analyzed by qRT-PCR. U6 was used as the internal control. (^&&&^*p* < 0.001 vs. normal) **B**. The level of miR-661 in NSCLC cells and normal cells was analyzed by qRT-PCR. U6 was used as the internal control. (^###^*p* < 0.001 vs.16HBE) **C**. The effects of circ_0001947 knockdown and miR- 661 inhibitor (I) on the level of miR-661 were analyzed by qRT-PCR. U6 was used as the internal control. (***p < 0.001 vs. si-NC + inhibitor control (IC) group; ^△△△^*p* < 0.001 vs. small interfering RNA targeting circ_0001947 (si-circ_0001947#2) + IC group; ^‡‡^ *p* < 0.01 vs. si-NC + I group) **D**. The effects of circ_0001947 overexpression and miR-661 mimic (M) on the level of miR-661 were analyzed by qRT-PCR. U6 was used as the internal control. (^^^*p* <0.001 vs. NC + MC group; ^†††^*p* <0.001 vs. NC + M group; ^ΦΦΦ^ *p* <0.001 vs. circ_0001947 + MC group) **E**. The effects of circ_0001947 knockdown and miR-661 I on cell viability were analyzed by CCK-8. (*p < 0.05, ** *p* < 0.01 vs. si-NC + IC group; ^△^*p* <0.05 vs. si-circ_0001947#2 + IC group; ^‡^ *p* < 0.05 vs. si-NC + I group) **F**. The effects of circ_0001947 overexpression and miR-661 M on cell viability were analyzed by CCK-8. (^*p* < 0.05, ^^^ *p* < 0.001 vs. NC + MC group; ^ΦΦ^ *p* < 0.01, ^ΦΦΦ^ *p* < 0.001 vs. circ_0001947 + MC group; ^†^*p* < 0.05 vs. NC + M group)

Our study revealed that miR-661 was overexpressed in NSCLC tissues and cells (Figs. 4A and 4B). Furthermore, we explored the role of miR-661 in the regulation of circ_0001947 on the viability of H1299 and A549 cells. Interestingly, the miR-661 level was intensified by si-circ_0001947#2 but was weakened by miR-661 I, while the above effects were overturned by co-transfection of si-circ_0001947#2 and miR-661 I (Fig. 4C). Co-transfection of overexpressed circ_0001947 and miR-661 M reversed the regulation of miR-661 M / overexpressed circ_0001947 on the miR-661 level (Fig. 4D). Moreover, cell viability was elevated by si-circ_0001947#2 but was inhibited by miR-661 I, the two tendencies were reversed by co-transfection of si-circ_0001947#2 and miR-661 I (Fig. 4E). However, co-transfection of overexpressed circ_0001947 and miR-661 M partially offset the regulation of miR-661 M / overexpressed circ_0001947 on cell viability (Fig. 4F).

### MiR-661 Was Involved in the Regulation of circ_0001947 on Apoptosis, Proliferation, and Cell Cycle of NSCLC Cells

**Fig. 5:**
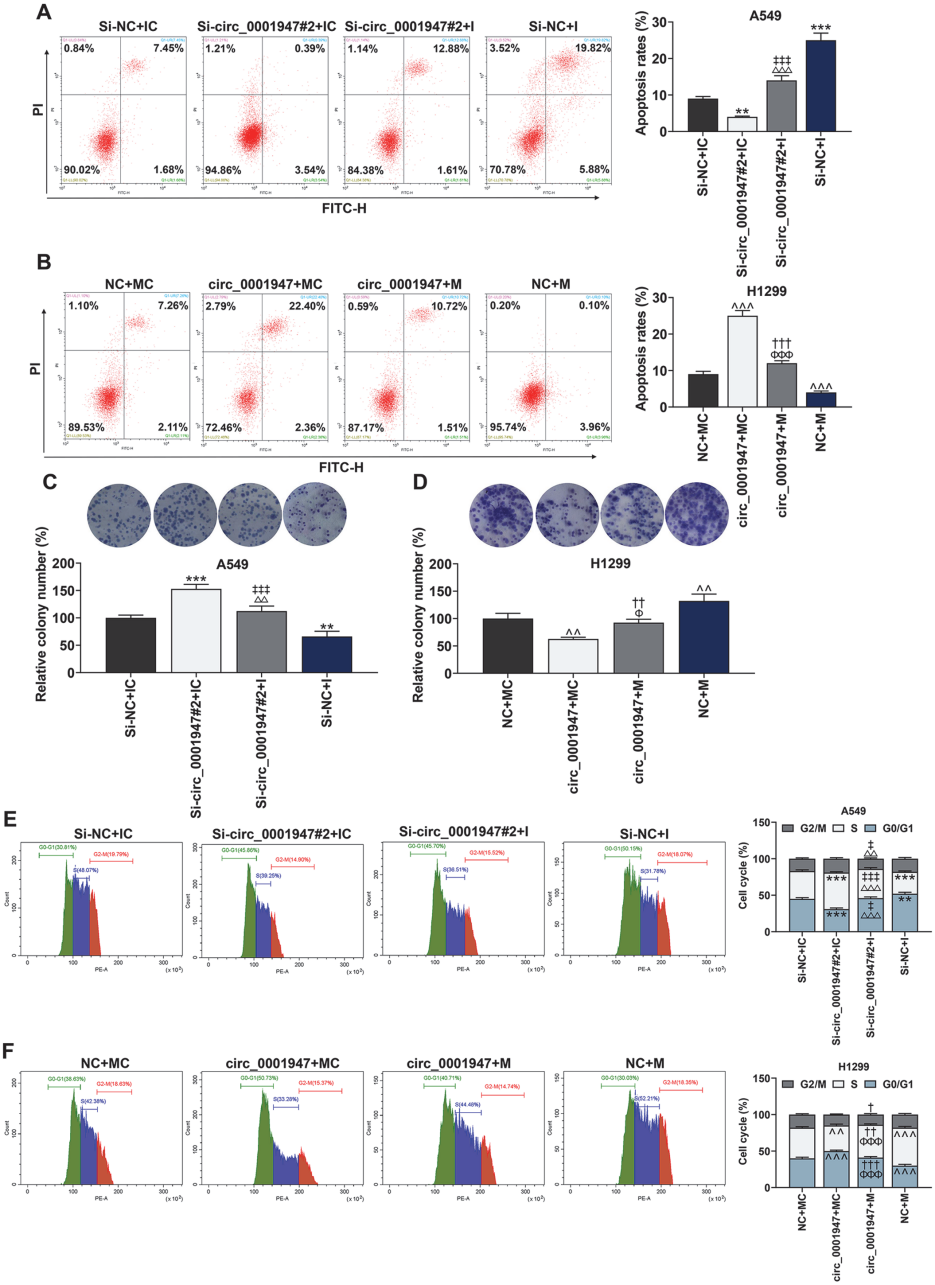
MiR-661 was involved in the regulation of circ_0001947 on apoptosis, proliferation, and cell cycle of NSCLC cells. **A**. The effects of circ_0001947 knockdown and miR-661 I on apoptosis were analyzed by flow cytometry. (**p < 0.01, *** *p* < 0.001 vs. si-NC + IC group; ^‡‡‡^*p* < 0.001 vs. si-NC + I group; ^△△△^*p* < 0.001 vs. si-circ_0001947#2 + IC group) **B**. The effects of circ_0001947 overexpression and miR-661 M on apoptosis were analyzed by flow cytometry. (^^^*p* < 0.001 vs. NC + MC group; ^ΦΦΦ^*p* < 0.001 vs. circ_0001947 + MC group; ^†††^*p* <0.001 vs. NC + M group) **C**. The effects of circ_0001947 knockdown and miR-661 I on cell proliferation were analyzed by colony formation assay. ** *p* < 0.01, *** *p* < 0.001 vs. si-NC + IC group; ^‡‡‡^ *p* < 0.001 vs. si-NC + I group; ^△△^ *p* < 0.01 vs. si-circ_0001947#2 + IC group. **D**. The effects of circ_0001947 overexpression and miR-661 M on cell proliferation were analyzed by colony formation assay. (^^*p* < 0.01 vs. NC + MC group; ^††^*p* < 0.01 vs. NC + M group; ^Φ^ *p* < 0.05 vs. circ_0001947 + MC group) **E**. The effects of circ_0001947 knockdown and miR-661 I on cell cycle were analyzed by flow cytometry. (**p < 0.01, *** *p* < 0.001 vs. si-NC + IC group; ^‡^ *p* < 0.05, ^‡‡‡^ *p* <0.001 vs. si-NC + I group; ^△△^ *p* < 0.01, ^△△△^ *p* < 0.001 vs. si-circ_0001947#2 + IC group) **F**. The effects of circ_0001947 overexpression and miR-661 M on cell cycle were analyzed by flow cytometry. (^^*p* < 0.01, ^^^ *p* < 0.001 vs. NC + MC group; ^†^*p* < 0.05, ^††^*p* < 0.01, ^†††^*p* < 0.001 vs. NC + M group; ^ΦΦΦ^ *p* < 0.001 vs. circ_0001947 + MC group)

As delineated in Fig. 5A, the results unveiled that apoptosis was largely elevated by miR-661 I but was suppressed by si-circ_0001947#2, which was mitigated by co-transfection of si-circ_0001947#2 and I. Meanwhile, an increase in apoptosis induced by overexpressed circ_0001947 or a decrease in apoptosis triggered by miR-661 M was reversed by co-transfection of overexpressed circ_0001947 and miR-661 M (Fig. 5B). Then, we also proved that co-transfection of si-circ_0001947#2 and miR-661 I neutralized the inhibition of miR-661 I and the promotion of si-circ_0001947#2 on cell proliferation (Fig. 5C). Moreover, co-treatment of overexpressed circ_0001947 and miR-661 M partially offset the regulation of miR-661 M / overexpressed circ_0001947 on cell proliferation (Fig. 5D). The detection of cell cycle confirmed that si-circ_0001947#2 and miR-661 M induced cell cycle arrest at S phase, while miR-661 I and overexpressed circ_0001947 facilitated the entry of G0/G1 phase (Figs. 5E and 5F). Besides, the effect of si-circ_0001947#2 or miR-661 I on cell cycle was partially reversed by co-transfection of si-circ_0001947#2 and I , while the effect of overexpressed circ_0001947 or miR-661 M was partially reversed by co-transfection of overexpressed circ_0001947 and miR-661 M (Figs. 5E and 5F).

### MiR-661 Targeted DOK7

**Fig. 6:**
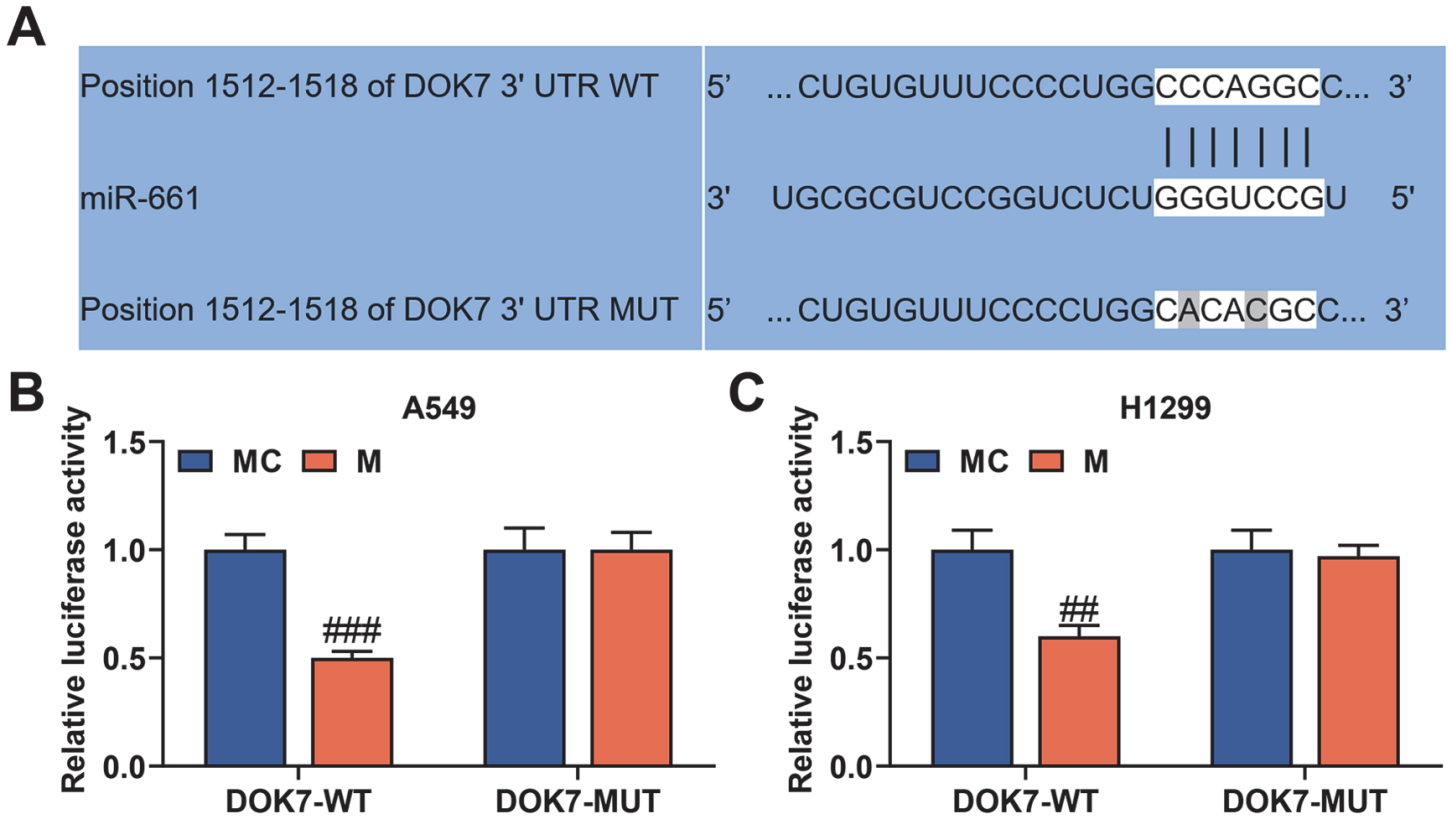
MiR-661 targeted DOK7. **A**. The binding sequences of DOK7 containing the miR-661 binding sites were determined by TargetScan v7.2 (https://www.targetscan.org/). **B-C**. Direct interaction between DOK7 and miR-661 was detected by dual-luciferase reporter assay. (^##^*p* < 0 .01, ^###^ *p* < 0.001 vs. MC group)

We used TargetScan v7.2 analysis to discover that miR-661 targeted DOK7 (Fig. 6A). Additionally, the relative luciferase activity was lower in the miR-661 M group than that in the miR-661 MC group when the sequence of DOK7 was wild type (Figs. 6B and 6C), while there was no obvious difference between miR-661 M and MC groups when the sequence of DOK7 was mutant.

### The Expression of DOK7 Was Down-Regulated in NSCLC Tissues and Cells, Which Was Regulated by Circ_0001947

**Fig. 7:**
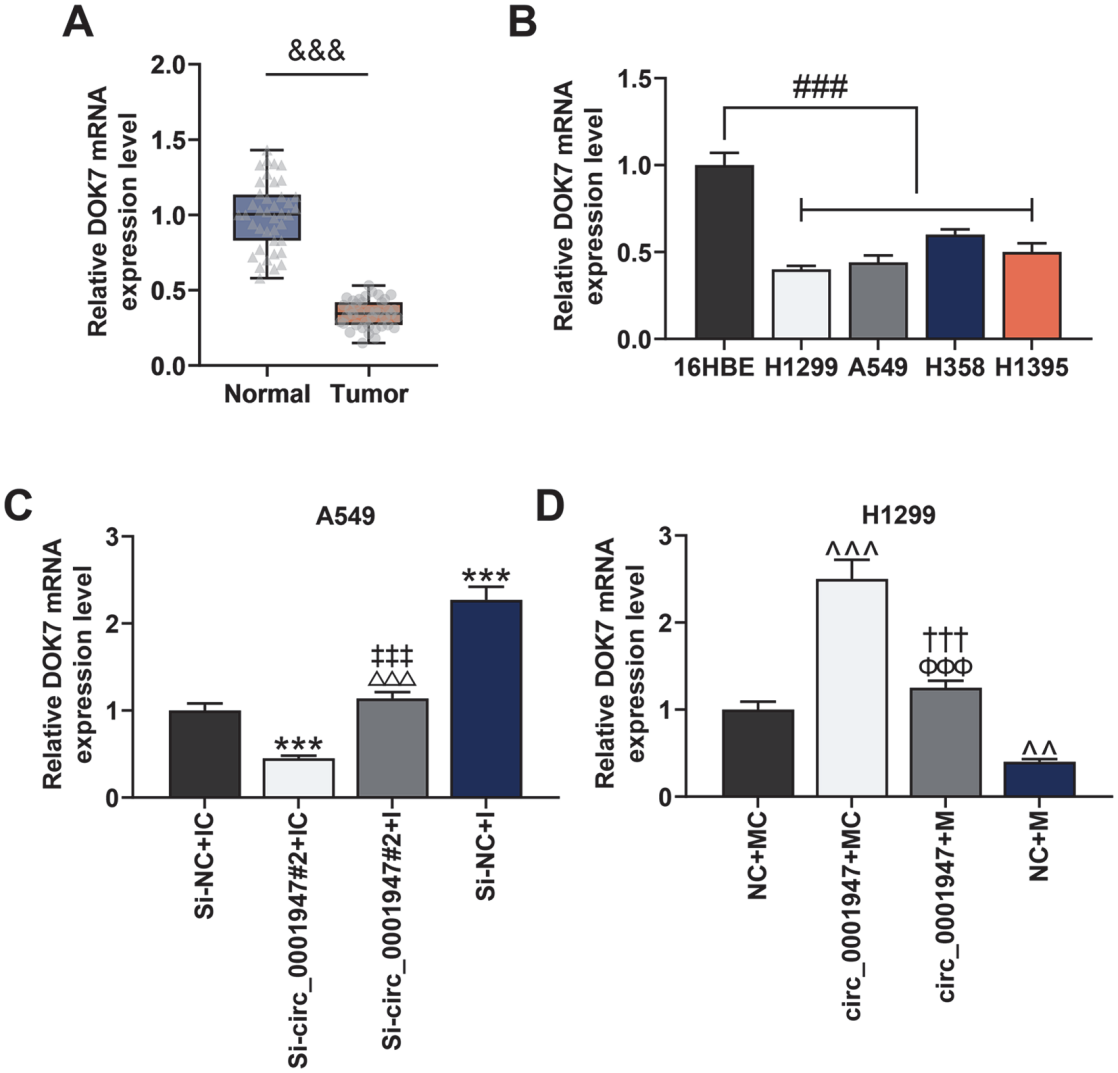
DOK7 was down-expressed in NSCLC tissues and cells, whose expression was regulated by circ_0001947. **A**. The level of DOK7 in 40 NSCLC tissues and adjacent non-tumor tissues was analyzed by qRT-PCR. GAPDH was used as the internal control. (^&&&^*p* < 0.001 vs. normal) **B**. The level of DOK7 in NSCLC cells and normal cells was analyzed by qRT-PCR. GAPDH was used as the internal control. (^###^*p* < 0.001 vs.16HBE) **C**. The effects of circ_0001947 knockdown and miR-661 I on DOK7 expression were analyzed by qRT-PCR. GAPDH was used as the internal control. (***p < 0.001 vs. si-NC + IC group; ^‡‡‡^ *p* < 0.001 vs. si-NC + I group; ^△△△^ *p* < 0.001 vs. si-circ_0001947#2 + IC group) **D**. The effects of circ_0001947 overexpression and miR-661 M on DOK7 expression were analyzed by qRT-PCR. GAPDH was used as the internal control. (^^*p* < 0.01, ^^^*P*<0.001 vs. NC + MC group; ^ΦΦΦ^*p* < 0.001 vs. circ_0001947 + MC group; ^†††^p < 0 .001 vs. NC + M group)

In this study, we first tested the level of DOK7 in NSCLC tissues or cell lines and manifested its downregulation (Figs. 7A and 7B). Next, we uncovered that DOK7 expression was dramatically inhibited by si-circ_0001947#2 and miR-661M, but was greatly promoted by overexpressed circ_0001947 and miR-661 I (Figs. 7C and 7D). Additionally, the effect of si-circ_0001947#2 or miR-661 I on the mRNA expression of DOK7 was partially reversed by co-transfection of si-circ_0001947#2 and miR-661 I in A549 cells, while the effect of overexpressed circ_0001947 or miR-661 M on the mRNA expression of DOK7 was partially reversed by co-transfection of overexpressed circ_0001947 and miR-661 M in H1299 cells (Figs. 7C and 7D).

### DOK7 Was Partly Involved in the Regulation of miR-661 on NSCLC Cell Viability

**Fig. 8:**
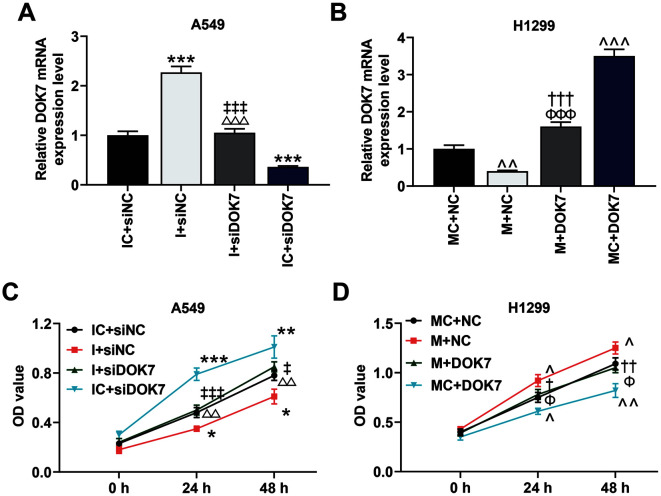
DOK7 was partly involved in the regulation of miR-661 on NSCLC cell viability. **A**. The effects of miR-661 I and small interfering RNA targeting DOK7 (siDOK7) on DOK7 expression were analyzed by qRT-PCR. GAPDH was used as the internal control. (***p < 0.001 vs. IC + siNC group; ^△△△^ *p* < 0.001 vs. I + siNC group; ^‡‡‡^ *p* < 0.001 vs. IC + siDOK7 group) **B**. The effects of miR-661 M and overexpressed DOK7 on DOK7 expression were analyzed by qRT-PCR. GAPDH was used as the internal control. (^^*p* < 0.01, ^^^ *p* < 0.001 vs. MC + NC group; ^†††^*P*<0.001 vs. MC + DOK7 group; ^ΦΦΦ^ *p* < 0.001 vs. M + NC group) **C**. The effects of miR-661 I and siDOK7 on cell viability were analyzed by CCK-8. (*p < 0.05, ** *p* < 0.01, *** *p* < 0.001 vs. IC + siNC group; ^△△^*p* < 0.01 vs. I + siNC group; ^‡^ *p* < 0.05, ^‡‡‡^ *p* < 0.001 vs. IC + siDOK7 group) **D**. The effects of miR-661 M and overexpressed DOK7 on cell viability were analyzed by CCK-8. (^*p* < 0.05, ^^ *p* < 0.01 vs. MC + NC group; ^†^*p* < 0.05, ^††^*p* < 0.01 vs. MC + DOK7 group; ^Φ^ *p* < 0.05 vs. M + NC group)

To figure out the role of DOK7 in the regulation of miR-661 on H1299 and A549 cells, we measured the alteration of DOK7 expression. Co-transfection of miR-661I and siDOK7 partially reversed the regulation of miR-661 I or siDOK7 on DOK7 expression (Fig. 8A). In the meantime, the effect of miR-661 M on restraining DOK7 expression was discovered to be mitigated by overexpressed DOK7 (Fig. 8B). Besides, the promotion of cell viability induced by siDOK7 and the inhibition of that induced by miR-661 I were reversed by co-transfection of miR-661 I and siDOK7 (Fig. 8C). The enhancement of miR-661 M on cell viability was offset by overexpressed DOK7 (Fig. 8D).

## Discussion

In our study, we tested the expression of circ_0001947 in NSCLC tissues, adjacent non-tumor tissues, and multiple NSCLC cell lines. Subsequently, we observed changes in cell viability, proliferation, apoptosis, and miRNAs expressions after circ_0001947 overexpression or silencing in H1299 and A549 cells. On this basis, we further verified the interactions between circ_0001947 and miR-661 as well as between miR-661 and downstream target genes, suggesting that miR-661 and downstream target genes might be the mediators of circ_0001947 in regulating the biological behaviors of NSCLC cells. Our findings revealed the role of circ_0001947 in modulating certain vital features of NSCLC.

First of all, we have clarified that circ_0001947 was low-expressed in NSCLC tissues and cell lines. Moreover, early research has demonstrated that circ_0001947 facilitates gastric cancer progression via modulating the miR-6894-5p/ANTXR1 axis [21]. Han *et al*. has put forward that circ_0001947 restrains acute myeloid leukemia development by modulating the miR-329-5p/CREBRF axis [12]. Our study complemented the research deficiency of circ_0001947 in NSCLC, which expounded for the first time that circ_0001947 overexpression inhibited cell viability and proliferation, induced cell cycle arrest at G0/G1 phase, and promoted apoptosis, while circ_0001947 knockdown exerted the opposite effects, manifesting the involvement of circ_0001947 in the progression of NSCLC.

Additionally, by searching the online database CircInteractome, we detected miR-510, miR-587, miR-661, and miR-942 expressions, whose functions and expressions were related to NSCLC. The qRT-PCR assay unearthed that circ_0001947 knockdown and overexpression made profound impacts upon the level of miR-661, and miR-661 was therefore chosen for follow-up experiments. CircRNAs are important members of the ceRNA network, playing crucial roles as "miRNA sponges" in the "circRNA-miRNA-mRNA" axis [22]. In our research, to probe into potential downstream pathways that may be implicated in the function of circ_0001947 in NSCLC, we performed database analysis and finally discovered that miR-661 may be a potential target of circ_0001947 which was further supported by the dual-luciferase reporter assay. MiR-661 has been confirmed to be abnormally expressed in a variety of malignant tumors and closely associated with the initiation and progression of tumors [23, 24]. Wang, *et al*. has disclosed that miR-661 is overexpressed in NSCLC tissues and cells and it accelerates NSCLC development via targeting RUNX3, which is consistent with findings of our research [25]. Liu *et al*. has proposed that miR-661 facilitates the invasion and metastasis of NSCLC cells via restraining RB1 [26]. Then, we provided the first demonstration that miR-661 inhibitor suppressed cell viability and proliferation, induced cell cycle arrest at G0/G1 phase, and elevated apoptosis, while the above effects were overturned by co-transfection of circ_0001947 knockdown and miR-661 inhibitor. It was clarified that miR-661 might serve as a regulator in the regulation of circ_0001947 on the cell biological behaviors of NSCLC cells.

Furthermore, in order to explore the target of miR-661 in NSCLC, we used database prediction software to predict the target gene of miR-661 and confirmed that DOK7 might be a potential target of miR-661. Luciferase assay corroborated that miR-661 could directly bind to 3’-UTR of DOK7. The DOK7 gene, a member of the DOK family, is located at the end of the short arm of chromosome 4 [27]. Recent studies have revealed that the DOK7 gene and its family are closely related to the initiation and development of many malignant tumors, such as lung cancer, breast cancer, and leukemia [28-30], but the detailed mechanism has not been fully understood. At present, there are few studies on the specific role of DOK7 in NSCLC. Only one study confirmed that DOK7 was greatly under-expressed in lung cancer, and DOK7 overexpression suppressed the proliferation and migration of lung cancer cells [27]. Consistent with the previous study, our data proved that DOK7 was down-expressed in NSCLC tissues and cells, the expression of which was discovered to be elevated by circ_0001947 overexpression through targeting miR-661. Meanwhile, DOK7 was partly involved in the regulation of miR-661 on NSCLC cell viability. It suggested that circ_0001947-modulated miR-661 could further modulate the upregulation of DOK7 in NSCLC cells.

However, despite the evidence that DOK7 is the target gene of miR-661, the upstream and downstream pathways of DOK7 are not tested, which may be a defect of this study. In addition, this study lacks in vivo experiments, which will be listed in our future experimental projects, so as to further improve the accuracy of this research.

In conclusions, altogether, this research emphasized that circ_0001947 acted as a sponge of miR-661 to restrain NSCLC progression via modulating DOK7, revealing that circ_0001947 might be a potential new biomarker for the treatment of NSCLC.

## Figures and Tables

**Table 1:** Gene sequence primers.

Name	Forward primer(5'-3')	Reverse primer(5'-3')
circ_0001947	ACACTCTTGGATGGAAAACCCA	CGTGTTCTGGACTCGGTTGG
miR-510	CTTCCATACTCAGGAGAGTGGC	TATCTCCAGACCAAGAC
miR-587	CCAGGCAAGAGAGAGTTGCTG	ATGGGCTTTCCACTGGTGATG
miR-661	AATGGTGGGTGCAAATGTGG	GAAACGCATGCCAAAAAGAC
miR-942	GCGCGCTCTTCTCTGTTTTGGC	GTGCAGGGTCCGAGGT
DOK7	GACAAGTCGGAGCGTATCAAG	ATGTCCTCTAGCGTCAGGCT
U6	GATTATCGGGACCATTCCACTG	GATCTGGTTCCCAATGACTGTG
GAPDH	AATGGATTTGGACGCATTGGT	TTTGCACTGGTACGTGTTGAT
